# Potential Role of Autonomic Dysfunction in Covid-19 Morbidity and Mortality

**DOI:** 10.3389/fphys.2020.561749

**Published:** 2020-10-16

**Authors:** Rodrigo Del Rio, Noah J. Marcus, Nibaldo C. Inestrosa

**Affiliations:** ^1^Laboratory of Cardiorespiratory Control, Department of Physiology, Pontificia Universidad Católica de Chile, Santiago, Chile; ^2^Department of Cell and Molecular Biology, Centro de Envejecimiento y Regeneración (CARE), Pontificia Universidad Católica de Chile, Santiago, Chile; ^3^Centro de Excelencia en Biomedicina de Magallanes (CEBIMA), Universidad de Magallanes, Punta Arenas, Chile; ^4^Department of Physiology and Pharmacology, Des Moines University, Des Moines, IA, United States

**Keywords:** autonomic nervous system, COVID-19, inflammation, sympathetic nervous system, parasympathetic, sympathetic reactivity

## Introduction

In December 2019, a novel coronavirus-related disease (Covid-19) emerged in Wuhan, China, caused by the severe acute respiratory syndrome corona virus 2 (SARS-CoV-2). This outbreak rapidly spread throughout the world, being declared a pandemic by the world health organization on March 11, 2020. Covid-19 and other coronavirus diseases such as SARS and MERS cause serious morbidity and mortality through initiation of severe viral pneumonia often progressing to acute respiratory distress syndrome (ARDS) and multiple organ system dysfunction (MODS) (Paules et al., 2020). Non-respiratory co-morbidities such as cardiac dysfunction, renal failure, and neurological abnormalities are reported in the literature; however it is still unclear whether these are a direct result of SARS-Cov-2 invading and destroying these tissues or are more broadly related to hypoxemia, inflammation, and/or thrombosis associated with ARDS/MODS in Covid-19 patients.

Currently, there are no approved antiviral therapies or vaccines available to combat Covid-19, however major efforts are ongoing worldwide to rapidly address this urgent need. Biomedical research has become fundamental in the understanding of the mechanism(s) by which SARS-CoV-2 infects its host and leads to deterioration of human health. So far, genetic analysis has revealed several similarities between SARS-CoV-2 and the virus that caused the SARS epidemic in 2002, including the molecular targets that SARS coronaviruses use to bind to their host cells (Hoffmann et al., 2020). Specifically, the SARS-CoV-2 “spike” protein binds to the type 2 angiotensin converting enzyme (ACE2) at the surface of the host cell (Hoffmann et al., 2020). ACE2 is widely expressed in the human body (found in lung, bowel, kidney, brain, blood vessels) and participates in the regulation of angiotensin II (ANGII), a potent vasoactive peptide hormone. Synthesis of ANGII depends on the catalytic activity of the type 1 angiotensin converting enzyme (ACE) and ANGII levels are modulated by the catalytic activity of ACE2 which hydrolyzes ANGII to angiotensin 1-7 (Díaz et al., 2020; Guan et al., 2020; Hoffmann et al., 2020; Huang et al., 2020; Paules et al., 2020; South et al., 2020; Yang et al., 2020), a by-product of ANGII cleavage. Thus, ACE2 activity serves to limit ANGII production and protect from the potentially damaging consequences of high levels of systemic ANGII (South et al., 2020). ACE2 is also associated with processes that attenuate inflammation, making this enzyme an interesting pharmacological target for disease processes that result in over-activation of inflammatory pathways (South et al., 2020).

In early March 2020, the first epidemiological and clinical characteristics of Covid-19 patients (191 patients) were published (Huang et al., 2020). Of the total number of patients confirmed with Covid-19, 48% of total hospitalized patients in this study had significant comorbidities including hypertension (30%), diabetes (19%), and heart disease (8%). A second study with 1,099 Covid-19 patients reported that the most severe outcomes were observed in patients with hypertension, diabetes mellitus, coronary heart disease, and cerebrovascular disease (Guan et al., 2020). In a third study, out of 140 patients positive for Covid-19, 30% had hypertension and 12% had diabetes. Finally, a study by Yang et al. (2020) found that in a cohort of patients who succumbed to Covid-19, the most prevalent comorbidities were diabetes and cardiovascular disease. Of note, most of the comorbidities reported in these studies are treated with ACE inhibitors (ACEi), as increased ANGII levels are a common pathophysiological feature. Recent opinion pieces have noted that in pre-clinical studies ACEi cause up-regulation of ACE2 and theorize that this could increase susceptibility of patients currently being treated with ACEi to infection by SARS-CoV-2. This observation has led some to hypothesize that withdrawing ACEi treatment in patients with hypertension or other cardio-metabolic conditions characterized by upregulation of the renin-angiotensin system (RAS) may decrease the risk of developing severe Covid-19. While this hypothesis is worthy of further study, to our knowledge there is no evidence at this time to support removal of ACEi or other RAS-related drugs in patients for whom they are indicated. In fact, we wish to advance the hypothesis that doing so could be directly detrimental to Covid-19 related morbidity in the cohort of patients who are statistically most likely to suffer the worst outcomes. There are well-established relationships between RAS activation and autonomic dysfunction in cardio-metabolic diseases such as hypertension, heart failure, and diabetes (Díaz et al., 2020). In these conditions often a positive feedback loop exists between RAS activation and tonic increases in efferent sympathetic nerve activity, wherein increases in sympathetic activity can stimulate activation of RAS which in turn can further upregulate sympathetic activity (Díaz et al., 2020). Removal of drugs which attenuate this positive feedback loop could in fact exacerbate autonomic dysfunction and inflammation associated with these diseases, and potentially contribute to morbidity associated with Covid-19.

## Autonomic Dysfunction and COVID-19 Morbidity and Mortality

Hypertension, heart failure, type II diabetes mellitus, and chronic kidney disease are all conditions associated with heightened sympathetic nerve activity, which in many cases contributes to disease progression (DiBona, 2013; Carnagarin et al., 2019; Díaz et al., 2020). Indeed, the existence of a pathophysiological continuum in these diseases, which encompasses increased sympathetic outflows, may predispose to further increases in sympathetic activity during Covid-19 infection. Accordingly, we propose that this common characteristic may be one important factor that contributes to higher morbidity and mortality after Covid-19 infection in patients with pre-existing conditions. Aside from the potential effects of these conditions (and their treatments) on ACE2 receptor expression and susceptibility to SARS-CoV-2 infection, these pre-existing conditions likely exacerbate cardiovascular complications associated with Covid-19. In the early phases of many cardio-metabolic conditions sympatho-excitation acts as an adaptive response, however over prolonged periods of time it ultimately becomes maladaptive and contributes to progressive declines in cardiac function (Díaz et al., 2020). Hyper-activation of brainstem pre-sympathetic neurons and increased sympathetic nerve activity in hypertension and heart failure contribute to creation of a pro-arrhythmic substrate and indeed are associated with higher incidence of ventricular arrhythmias (Díaz et al., 2020). In Covid-19 patients, with a pre-existing conditions characterized by excessive sympathetic activity (i.e., hypertension, diabetes, heart disease), the combination of hypoxemia associated with Covid-19 related pneumonia/ARDS, diffuse inflammation, and heightened sympathetic activity would increase susceptibility to lethal cardiac arrhythmias. These interrelationships, if proven, have the potential to trigger a vicious cycle contributing to worse outcomes in Covid-19 patients. Clinical data is still scarce in this area as a result of the short time frame from the start of the pandemic to the current time, however anecdotal reports from EMS indicate 10-fold increases in cardiac arrest calls in New York City over the same time period in years past. From such preliminary data it is difficult to discern the extent to which clotting disorders (also reported in Covid-19) and acute myocardial infarction (AMI) contributed to this marked rise in incidence of cardiac arrest calls. However, our hypothesis offers a plausible explanation for this increase in both the presence and absence of thrombosis and AMI. In addition to the effects on arrhythmic substrate in Covid-19 patients, autonomic dysfunction may exacerbate Covid-19 morbidity in other ways.

The vagal anti-inflammatory reflex is characterized by activation of a vagal cholinergic efferent arm in response to inflammation (Tracey, 2002). Activation of the vagal anti-inflammatory reflex results in the inhibition of cytokine synthesis by macrophages of the mononuclear phagocytic system including resident and circulating macrophages. These changes are observed in many tissues including the lungs, heart and brain. The presence of reciprocal inhibition in the autonomic nervous system suggests that sympatho-excitation is closely linked to parasympathetic (vagal) withdrawal (Díaz et al., 2020). Therefore, in pathological conditions such as hypertension and diabetes, it is plausible to hypothesize that a decrease in the neuro-vagal inflammatory reflex would occur and could contribute to loss of normal restraint of inflammatory processes. In Covid-19 patients, the so-called *cytokine-storm* is hypothesized to contribute to a fast transition from a compensated state to decompensated state requiring supplemental oxygen and/or mechanical ventilation. Accordingly, Covid-19 patients with parasympathetic withdrawal due to comorbidities, may be at higher risk of developing sudden increases in cytokine release due to the lack of a fully functional neuro-vagal anti-inflammatory reflex. In pre-clinical testing, vagal nerve stimulation leads to restoration of autonomic balance and reduction in pro-inflammatory cytokine levels in ischemic heart disease (De Ferrari et al., 2017). Theoretically, vagal stimulation could be a useful tool to address systemic inflammation by activating the neural inflammatory reflex in Covid-19 patients with pre-existing cardio-metabolic conditions characterized by autonomic imbalance.

Finally, it is important to mentioned that psychological stress after being diagnosed with Covid-19 may also contribute to autonomic dysfunction. Furthermore, emotional components associated with clinical isolation after hospital admission may hasten sympathoexcitation adding more stress to a failing heart due to SARS-CoV-2 infection. Also, patient families may be also prone to develop autonomic dysregulation since they need to cope with emotional distress. Together, these arguments require to be taken into account when addressing autonomic nervous system outcomes during the present Covid-19 pandemic.

## Summary and Conclusion

**Figure 1:**
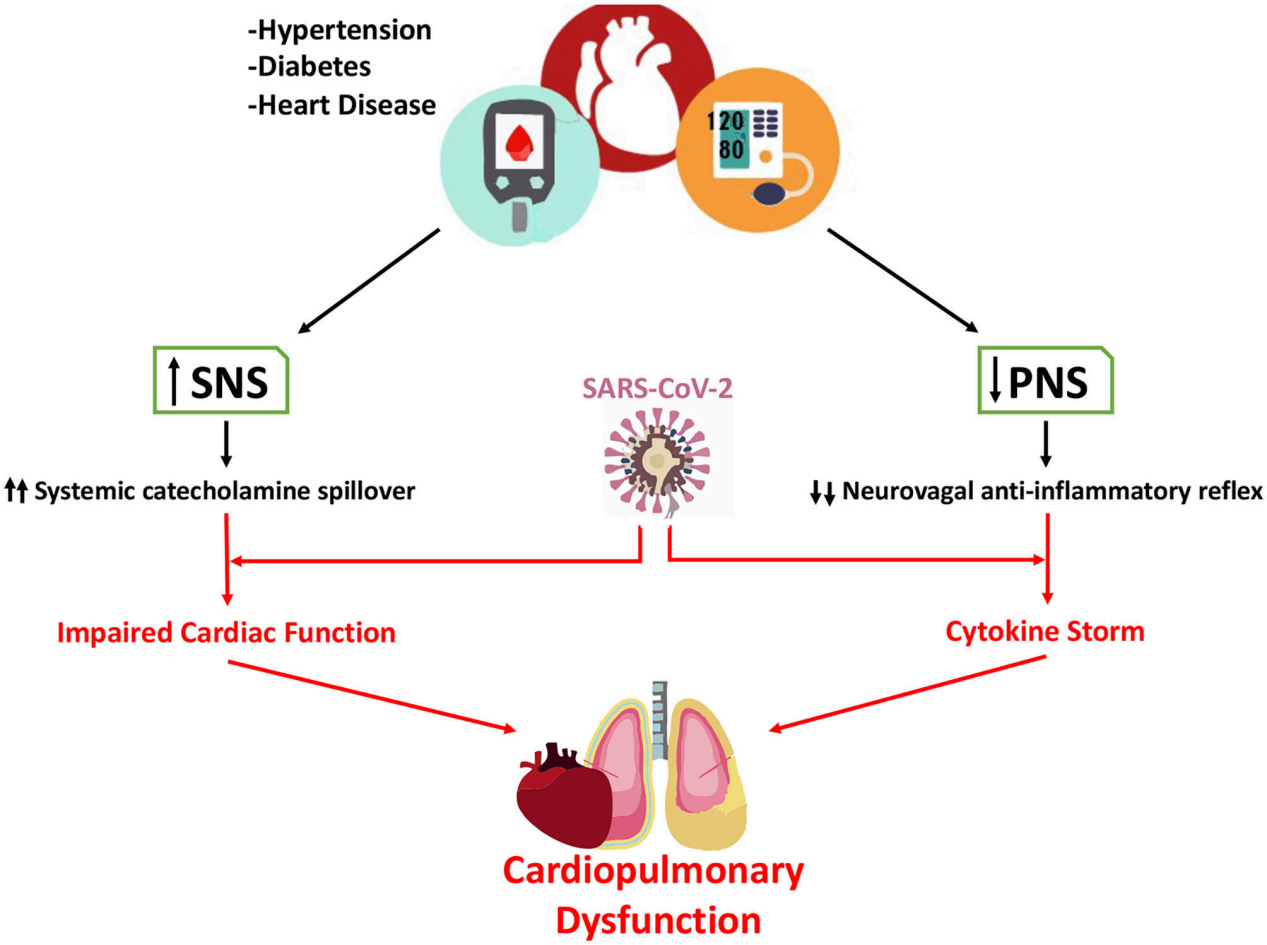
Contribution of altered autonomic function in hypertension, diabetes, and heart disease to poor outcomes in Covid-19 patients. It is well-known that sympathetic nerve activity is significantly increase in patients with hypertension, diabetes, and heart disease. In contrast, parasympathetic activity is markedly decreased in these patients. In Covid-19 patients both sympatho-excitation and parasympathetic withdrawal may play a pivotal role in increasing the risk of life-threating events. Enhanced sympathetic activity directly increases circulating catecholamines and increases myocardial work and oxygen demand, adding more stress to the Covid-19 heart. Simultaneously, the reduction in parasympathetic activity reduces the neuro-vagal anti-inflammatory reflex hastening the release of pro-inflammatory cytokines and potentially contributing to cytokine storm.

Autonomic dysfunction characterized by heightened sympathetic activity and withdrawal of parasympathetic activity is a common pathophysiological hallmark in patients with hypertension, diabetes and heart disease. Importantly, recent epidemiological data indicates that Covid-19 patients with these co-morbidities are at higher risk for developing life-threating complications. ACEi, a first-line drug used in treatment of these diseases has been suggested as a possible mediator of poor outcomes in Covid-19 patients. Treatment with ACEi may lead to the up-regulation of ACE2 gene, which is the molecular target that SARS-CoV-2 uses to infect the host cell. While this hypothesis has merit it is critical that strong and compelling experimental evidence is presented before considering withdrawal of ACEi in Covid-19 patients. In this brief letter, we have presented a complementary theory that could explain poor outcomes in Covid-19 patients with hypertension, diabetes and/or heart disease as comorbidities (Figure 1). Specifically, we hypothesized that enhanced resting sympathetic activity (normally occurring in hypertension, diabetes and heart disease) combined with hypoxemia would add significant stress to hearts already potentially beset by dysfunction and possibly viral myocarditis. The increased cardiac workload and decreased arterial oxygen content would lead to oxygen supply/demand mismatch in cardiac tissue and amplify existing pro-arrhythmic substrates. Decreases in parasympathetic activity occurring in tandem with sympatho-excitation may blunt the neuro-vagal anti-inflammatory reflex response during SARS-CoV-2 infection potentially contributing to massive cytokine release (i.e., cytokine storm). While there are likely many important aspects of the pathophysiology of Covid-19 in patients with poor outcomes, we strongly believe that the potential detrimental contribution of autonomic dysfunction merits further study. We strongly believe in the importance of clinical assessment of autonomic function in patients diagnosed with Covid-19, both during and after infection. Fortunately, there are several direct and indirect methods that are suitable to introduce in the clinic [for review see Zygmunt and Stanczyk (2010)]. Novel non-invasive imaging techniques could be a potent tool to assess activity in brain sympathetic/parasympathetic control areas in patients affected by Covid-19 (Macefield and Henderson, 2020). Including assessment of autonomic function in multicenter studies could aid in the determination of the impacts of autonomic dysfunction on Covid-19 morbidity as well as the potential effects of Covid-19 to generate novel autonomic dysfunction.

## Author Contributions

All authors listed have made a substantial, direct and intellectual contribution to the work, and approved it for publication.

## Conflict of Interest

The authors declare that the research was conducted in the absence of any commercial or financial relationships that could be construed as a potential conflict of interest.
